# Fcγ receptors are required for NF-κB signaling, microglial activation and dopaminergic neurodegeneration in an AAV-synuclein mouse model of Parkinson's disease

**DOI:** 10.1186/1750-1326-5-42

**Published:** 2010-10-26

**Authors:** Shuwen Cao, Shaji Theodore, David G Standaert

**Affiliations:** 1 Center for Neurodegeneration and Experimental Therapeutics, Department of Neurology, The University of Alabama at Birmingham, USA

## Abstract

Overexpression of alpha-synuclein (α-SYN), a protein which plays an important role in the pathogenesis of Parkinson's disease (PD), triggers microglial activation and adaptive immune responses, and leads to neurodegeneration of dopaminergic (DA) neurons. We hypothesized a link between the humoral adaptive immune response and microglial activation in α-SYN induced neurodegeneration. To test this hypothesis, we employed adeno-associated virus serotype 2 (AAV2) to selectively over-express human α-SYN in the substantia nigra (SN) of wild-type mice and FcγR-/- mice, which lack high-affinity receptors for IgG. We found that in wild-type mice, α-SYN induced the expression of NF-κB p65 and pro-inflammatory molecules. In FcγR-/- mice, NF-κB activation was blocked and pro-inflammatory signaling was reduced. Microglial activation was examined using immunohistochemistry for gp91PHOX. At four weeks, microglia were strongly activated in wild-type mice, while microglial activation was attenuated in FcγR-/- mice. Dopaminergic neurodegeneration was examined using immunohistochemistry for tyrosine hydroxylase (TH) and unbiased stereology. α-SYN overexpression led to the appearance of dysmorphic neurites, and a loss of DA neurons in the SN in wild-type animals, while FcγR-/- mice did not exhibit neuritic change and were protected from α-SYN-induced neurodegeneration 24 weeks after injection. Our results suggest that the humoral adaptive immune response triggered by excess α-SYN plays a causative role in microglial activation through IgG-FcγR interaction. This involves NF-κB signaling, and leads to DA neurodegeneration. Therefore, blocking either FcγR signaling or specific intracellular signal transduction events downstream of FcγR-IgG interaction, such as NF-κB activation, may be viable therapeutic strategies in PD.

## Background

Parkinson's disease (PD) is a neurodegenerative disease characterized primarily by loss of dopaminergic (DA) neurons in the substantia nigra (SN) of the midbrain. The protein alpha-synuclein (α-SYN) is closely linked to the pathogenesis of PD: genetic mutations or multiplication of the gene coding α-SYN, *SNCA*, cause familial forms of PD, while α-SYN is the main component of the protein aggregates, Lewy bodies and Lewy neurites found in sporadic PD [1-4]. Despite the abundant evidence for the role of α-SYN in the pathogenesis of PD, the mechanism by which excess α-SYN leads to neurodegeneration is still unknown.

Neuroinflammation is a constant feature of PD. Microglial activation is observed in the SN of PD patients [5] and there is a correlation between the extent of microglial activation in the SN and the degree of α-SYN accumulation [6]. In neurotoxin-induced animal models of PD, microglia are strongly activated after the administration of MPTP, rotenone or 6-OHDA [7-10], and inhibition of microglial activation in these models attenuates the toxin-related DA neurodegeneration [11,12]. While cell death is a prominent component of the toxin-mediated models of PD, it is not a necessary antecedent of inflammation. Our previous studies in a mouse model in which α-SYN expression is driven by an adeno-associated virus serotype 2 (AAV2) viral vector revealed that overexpression of human α-SYN leads to IgG deposition, classical microglial activation with increased production of pro-inflammatory cytokines, and B and T-lymphocyte infiltration in the SN long before overt neurodegeneration is apparent [13].

An important link between the innate immune system of the brain and the adaptive immune system, mediated by circulating B and T cells, is the family of Fc gamma receptors (FcγR). They are present on the surface of microglia as well as other cell types, including natural killer cells, neutrophils, and mast cells. Fcγ receptors bind immunoglobulin G (IgG) and trigger signal transduction events leading to microglial activation [14]. There is indirect evidence for the importance of Fcγ receptors in PD: in postmortem human brain, PD is associated with increased binding of IgG to DA neurons in SN and elevated levels of FcγR on microglia [15]; FcγRI-dependent microglial responses to IgG from PD patients have been demonstrated *in vitro *[16]; and loss of DA neurons is observed in the SN of rodents following injection of IgG purified from the sera of PD patients, a process which is also FcγR dependent [17,18].

Major downstream mediators of FcγR activation are the NF-κB class of transcription factors, which are important for the regulation of immune and inflammatory responses [19]. They are homo- or heterodimers composed of members of the NF-κB/Rel family, which include RelA (p65), RelB, cRel, p50 and p52 [20]. Promoter regions of many of the pro-inflammatory cytokines, which are elevated in neurodegenerative conditions, contain DNA binding sites for NF-κB family proteins, and the inhibition of NF-κB activation reduces the induction of pro-inflammatory molecules [21]. In human PD brain, there is evidence for increased expression and nuclear translocation of NF-κB p65 protein, and similar findings are seen in MPTP-intoxicated mice [22]. In the MPTP mouse model, injection of NEMO-binding domain (NBD) peptide, which inhibits NF-κB activation, suppresses microglial activation and cytokine production, and improves motor outcomes [22].

We hypothesized a critical role of microglial FcγR proteins in linking the humoral arm of the adaptive immune response to microglial activation and NF-κB signaling in α-SYN induced neuro-degeneration. To test this hypothesis, we used adeno-associated virus serotype 2 (AAV2) to selectively over-express human α-SYN in the substantia nigra (SN) of wild-type mice and in mice that lack the high-affinity receptors for immunoglobulin G (FcγR-/- mice), and examined the role of these receptors in coupling to neuroinflammation and neurodegeneration.

## Methods

### Animals and Treatment

Male C57BL/6 mice (8-12 weeks old) were used for the study. The FcγR-/- mice from C57BL/6 background were obtained from Taconic labs (model # 000583-M-M, Taconic, Hudson, NY, USA). These mice (nomenclature: B6.129P2-Fcer1g^tm1Rav^N12) are deficient in the gamma chain subunit of the FcγRI, FcγRIII and FcεRI receptors. They exhibit immune system defects such as inability to phagocytose antibody-coated particles, and the inflammatory responses to immune complexes are attenuated [23]. The AAV2 plasmids as well as the helper plasmid pDG-1 (a kind gift from Dr. Ponnazhagan, University of Alabama at Birmingham) were purified using cesium chloride density gradient ultracentrifugation. Recombinant AAV2, containing the gene for human α-SYN (AAV2-SYN) or green fluorescent protein (AAV2-GFP) was packaged by co-transfecting AAV2-SYN/GFP plasmids and pDG-1 into HEK-293 cells using a protocol previously described [24]. AAV2-SYN and AAV2-GFP were purified by a single step column purification protocol using heparin agarose columns [25]. Under isoflurane anesthesia, the mice were injected stereotaxically with 2 μl of AAV2-SYN (6.0 × 10^10 ^viral genome/ml) or AAV2-GFP (2.65 × 10^11 ^viral genome/ml) into the right SN; co-ordinates were anterior-posterior, -3.2 mm from bregma, medio-lateral, -1.2 from midline and dorso-ventral, -4.6 from the dura.

### Immunohistochemistry

Animals were sacrificed at 2 weeks, 4 weeks and 24 weeks following AAV2-SYN or AAV2-GFP injection and brain tissue was processed for immunohistochemistry. In the 2 and 4-week treatment groups, for examining NF-κB activation, we used anti-NF-κB p65 and anti-NF-κB p50 antibodies. For examining microglial activation, we used the marker gp91PHOX (a subunit of the enzyme NADPH-oxidase). For cell type identification, we used anti-CD68 antibody to detect microglia. Free-floating SN tissue sections (40μm thick) were incubated with rabbit anti-human-α-SYN (1:1000, Biosource, Camarillo, CA), rabbit anti-green fluorescent protein (1:1000, Abcam, Cambridge, MA), mouse anti-NF-κB p65 (1:100 Santa Cruz Biotechnology, Santa Cruz, CA), mouse anti-gp91PHOX (1:1000 AbD Serotec, Oxford, UK), rat anti-CD68 (1:500 Abcam, Cambridge, MA) or mouse anti-green fluorescent protein (1:500 Millipore, Billerica, MA), rabbit anti-NF-κB p50 (1:100 Santa Cruz Biotechnology, Santa Cruz, CA) antibodies followed by 1:500 dilution of alexa-488 conjugated goat anti-rabbit or anti-rat (Molecular probes, Carlsbad, CA) and 1:500 dilution of CY3-conjugated goat anti-mouse (Jackson Immunoresearch, West Grove, PA) antibodies. Since some antibodies were derived from a mouse host, the sections were first blocked with a goat anti-mouse F(ab_2_) fragment to prevent non-specific background staining by the secondary antibody.

In the 24-week treatment groups, the mice SN sections revealed significant autofluorescence and background staining upon examination with a fluorescence microscope, which interfered with accurate examination of the gp91PHOX immunostained microglia. Therefore, for this time point, we used diaminobenzidine combined with nickel sulfate intensification to visualize gp91PHOX positive microglia. Briefly, the SN sections were stained sequentially, first for gp91PHOX and developed with diaminobenzidine and nickel sulfate, followed by staining for SYN/GFP and developed with only diaminobenzidine. The primary antibody concentration was the same as above. The secondary antibodies used were peroxidase conjugated goat anti-mouse (1:2000, Jackson Immunoresearch, West Grove, PA) for gp91PHOX and peroxidase-conjugated goat anti-rabbit (1:2000, Jackson Immunoresearch, West Grove, PA) for SYN/GFP. For TH immunostaining, a rabbit polyclonal primary antibody (Pelfreeze Biologicals, Rogers, AR) was used at 1:2000 dilution. The secondary antibody used was a biotinylated goat anti-rabbit (1:4000, Vector Labs, Burlingame, CA) followed by horseradish peroxidase conjugated streptavidin (1:1000, Vector Labs, Burlingame, CA). The staining was developed using diaminobenzidine. For the detection of neuritic change, we used a mouse anti-TH antibody (1:1000, Sigma, St. Louis, MO) and rabbit anti-green fluorescent protein (1:1000, Abcam, Cambridge, MA) or rabbit anti-human-α-SYN (1:1000, Biosource, Camarillo, CA) antibodies followed by 1:500 dilution of alexa-488 conjugated goat anti-rabbit (Molecular probes, Carlsbad, CA) and 1:500 dilution of CY3-conjugated goat anti-mouse (Jackson Immunoresearch, West Grove, PA) antibodies.

### Imaging and Quantification

Confocal images were captured using a Leica TCS-SP5 laser scanning confocal microscope. The images were processed using the Leica software and exported as TIFF files and processed using Adobe Photoshop CS2. For quantitation of NFκB p65, NFκB p50 and gp91PHOX staining, the slides were observed using a Nikon Eclipse E800 M fluorescent microscope. Coded slides were scored using a numerical scale from 0 (no staining) to 4 (most intense) by an observer blind to the treatment paradigm. Only staining in close proximity to SN neurons was considered for scoring, while staining along the needle tract was ignored. Scores obtained from six mice per group were statistically analyzed using Mann-Whitney U test.

### Isolation of Nuclear Extracts from Midbrain Tissues and Immunoblotting

Ventral midbrains were frozen immediately on dry ice and nuclear extracts were obtained using NE-PER^® ^Nuclear and Cytoplasmic Extraction Reagents (Thermo Scientific, Rockford, IL). The nuclear extracts were resolved on 8% sodium dodecyl sulfate polyacrylamide gel. Proteins were transferred to nitrocellulose membranes incubated with mouse anti-NF-κB p65 (1:1000 Santa Cruz Biotechnology, Santa Cruz, CA) or rabbit anti-NF-κB p50 (1:1000 Santa Cruz Biotechnology, Santa Cruz, CA) antibodies followed by secondary (1:2000 Jackson Immunoresearch, West Grove, PA) antibodies labeled with horseradish peroxidase. Immunostained bands were detected using ECL kit (Thermo Scientific, Rockford, IL). Blots were normalized with lamin (1:1000 Cell Signaling Technology, Danvers, MA) as appropriate.

### Estimation of markers of NF-κB activation in the SN using quantitative PCR

Male C57BL/6 and FcγR-/- mice were injected stereotaxically with AAV2-SYN or AAV2-GFP into the right SN. Two weeks later, the animals were euthanized and the SN from the injected side was dissected out and stored at -80°C until assayed by quantitative PCR. Lipopolysaccharide (LPS, Sigma, St. Louis, MO) was injected into the right SN at a volume of 2 μl at 2.5 μg/μl concentration to a separate group of mice as a positive control. Total RNA from the injected SN was isolated using the TRI reagent (Sigma, St. Louis, MO) and purified using the RNeasy mini kit (Qiagen, Valencia, CA). The RNA was then reverse transcribed into cDNA using a superscript III kit (Invitrogen, Carlsbad, CA) and cDNA was measured spectrophotometrically and stored at -20°C. Primers for the markers were designed using the Primer3 program http://frodo.wi.mit.edu/. Quantitative-PCR was performed using a Bio-Rad IQ5 multicolor real time PCR system. 100 ng/μl of cDNA was used for the reaction. Serial dilutions of cDNA from LPS injected mice served as positive control and also as the source of standard curve from which the values for pro-inflammatory molecules were extrapolated. The expression levels of the markers examined were normalized against GAPDH mRNA. Expression ratios were analyzed statistically using one-way ANOVA. We studied markers of NF-κB activation (p65 and p50) as well as a typical marker for neuroinflammation, intercellular adhesion molecule 1 (ICAM-1). The markers and the primers used are listed in Table 1.

**Table 1:** Markers of NF-κB activation and their primers used for quantitative PCR

Markers	Forward Primer	Reverse Primer
NF-κB p65	GCGTACACATTCTGGGGAGT	GTTAATGCTCCTGCGAAAGC
NF-κB p50	CACCTAGCTGCCAAAGAAGG	GCAGGCTATTGCTCATCACA
ICAM-1	CAGCTACCATCCCAAAGCTC	CTTCAGAGGCAGGAAACAGG

### Stereological quantitation of dopamine neurons

TH immunoreactive dopamine neurons were quantified using unbiased stereology. Briefly, coded slides were scanned on the stage of a modified Olympus BX51 microbrightfield microscope under low-power objective, and SN on the injected and un-injected side were contoured. TH-positive neurons were counted on both sides by an optical fractionator method using stereoinvestigator 7.0 software from MBF Biosciences (Microbrightfield Inc, Williston, VT). A total of 4 sections covering the rostro-caudal extent of the SN around the injection site were counted and the number weighted section thickness was used to correct for variations in tissue thickness at different sites.

## Results

### AAV2-mediated overexpression of α-SYN triggers NF-κB activation with accumulation of p65 protein

**Figure 1:**
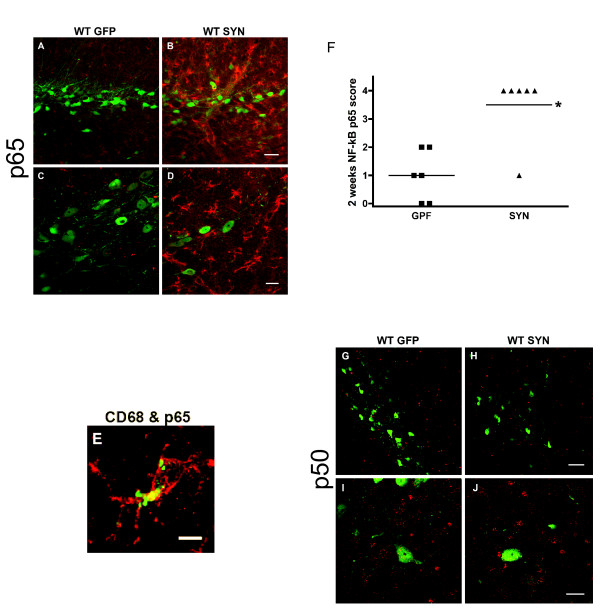
**Effect of over-expression of human α-SYN on NF-κB activation in the mouse SN at two weeks**. AAV2-SYN and AAV2-GFP (control) were injected into the right SN, and the tissue was processed for p65 and p50 staining at 2 weeks post-injection. A-D) The AAV2-SYN-injected SN displayed increased staining for p65 (red), mostly within microglia in close proximity to α-SYN-expressing neurons (green) while no induction of p65 was seen after AAV2-GFP. E) The staining for p65 (red) co-localized with the staining for CD68 (green), a marker for activated microglia, showing that NF-κB activation occurs in microglia. F) Scoring of immunostaining for p65 on a scale of 0 to 4. Compared with AAV2-GFP-injected tissues, AAV2-SYN treatment caused a significant increase in NF-κB p65. *p < 0.05 using Mann-Whitney U test (n = 6 per group). G-J) No significant difference was observed in NF-κB p50 immunoreactivity between AAV2-GFP and AAV2-SYN treated groups. Scale bars: panels A, B bar = 40 μm; panel E bar = 5 μm; panels G, H bar = 60 μm; panels C, D, I, J bar = 20 μm.

Groups of 6 of wild-type male C57BL/6 mice were injected stereotaxically with AAV2-SYN or AAV2-GFP into the right SN. Over-expression of human α-SYN resulted in robust expression of NF-κB p65 in mouse SN, while little or no activation was observed in AAV2-GFP treated mice two weeks after injection (Figure 1A-D). Examination of a separate set of animals injected only with PBS vehicle revealed similar low levels of p65 staining (not illustrated). In AAV2-SYN injected mice, NF-κB p65 expression was evident mainly in cells with the morphology of activated microglia, surrounding the human α-SYN-expressing neurons in SN, which was further confirmed by the co-localization of CD68 and NF-κB p65 staining (Figure 1E). For quantification of NF-κB p65 staining, coded slides stained for NF-κB p65 were scored using a numerical scale from 0 (no staining) to 4 (most intense) by an observer blinded to the treatment paradigm. This analysis confirmed the impression of markedly enhanced NF-κB p65 staining in the AAV2-SYN injected animals compared to the AAV2-GFP controls (Figure 1F).

A similar immunohistochemical analysis of the NF-κB p50 protein was performed in these animals. In contrast to the striking changes in p65, neither the AAV2-SYN nor the AAV2-GFP vector induced an appreciable enhancement of p50 staining over baseline (Figure 1G-J), and the scores assigned by the blinded observer did not differ significantly between the groups (not illustrated).

### FcγR-/- blocks NF-κB activation and p65 accumulation after AAV2-SYN

**Figure 2:**
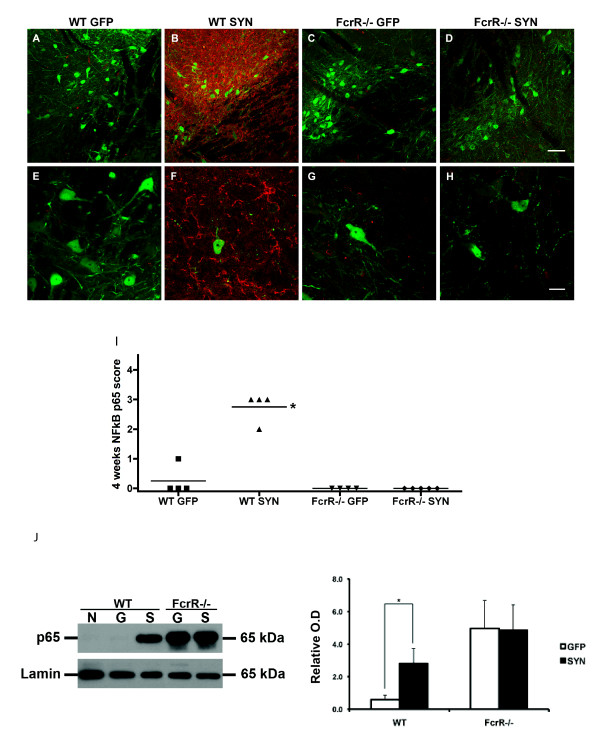
**Effect of α-SYN over-expression on NF-κB activation in wild-type and FcγR-/- mice at four weeks**. A-H) SN sections of wild-type and FcγR-/- mice over-expressing human α-SYN or GFP were double stained for NF-κB p65 (red) and SYN/GFP (green). Wild-type mice expressing human α-SYN revealed significantly increased immunoreactivity for NF-κB p65 while no significant enhancement of p65 was observed in FcγR-/- mice. Scale bars: panels A, B, C, D bar = 60 μm; panels E, F, G, H bar = 20 μm. I) Scoring of immunostaining for p65 using a rating scale revealed significant elevated p65 expression in wild-type mice treated with AAV2-SYN compared to AAV2-GFP group. This difference was not observed in FcγR-/- mice expressing SYN or GFP. *p < 0.05, WT-SYN vs WT-GFP, Mann-Whitney U test. J) Immunoblotting and quantification for nuclear NF-κB p65 in WT and FcγR-/- mice. In WT mice, AAV2-SYN treated mice showed significant increase in nuclear p65 level compared with AAV2-GFP controls. No difference was observed between FcγR-/- mice expressing α-SYN or GFP, but FcγR-/- mice have higher baseline levels of nuclear p65 than WT mice. *p < 0.05, WT-SYN vs WT-GFP t-test (N: untreated mice; G: AAV2-GFP mice; S: AAV2-SYN mice).

To study the role of FcγR in the activation of NF-κB by AAV2-SYN, we treated mice with unilateral injections of either AAV2-SYN or AAV2-GFP into the right SN. A matched group of male FcγR-/- mice and male wild-type controls (n = 4-5 in each treatment group) were studied four weeks after injection. In these animals, we observed that the expression of the α-SYN and GFP transgenes induced by the AAV2 vectors was similar in the FcγR-/- mice and the wild type mice. In the WT mice, there was strong activation of NF-κB p65 at 4 weeks, similar to that seen at 2 weeks in the previous experiment. Nevertheless, there was a striking difference in the expression of NF-κB p65 in the FcγR-/- mice injected with the AAV2-SYN vector. In contrast to the vigorous staining observed in the wild-type animals, AAV2-SYN treatment of the FcγR-/- mice produced no apparent accumulation of p65 (Figure 2A-H). This impression was confirmed by statistical analysis of p65 scoring performed by the blinded observer (Figure 2I).

To assess accurately the nuclear accumulation of NF-κB components, immunoblotting was performed with nuclear extracts of the midbrains from different treatment groups. In wild-type groups, there was significant increase of nuclear NF-κB p65 in AAV2-SYN treated mice compared with AAV2-GFP mice (Figure 2J). The FcγR-/- groups showed much higher baseline levels of nuclear NF-κB p65 than WT groups, however, within FcγR-/- groups, over-expresssion of human α-SYN did not lead to any further increase in NF-κB p65 activation compared with AAV2-GFP treated FcγR-/- controls (Figure 2J). For nuclear NF-κB p50 levels, there was no difference between AAV2-GFP and AAV2-SYN mice both in WT and FcγR-/- groups (not illustrated).

### FcγR-/- blocks transcriptional induction of NF-κB components and ICAM-1 after AAV2-SYN

The effects of FcγR deletion on the transcription of NF-κB components and the inflammatory mediator ICAM-1 was examined in a separate group of experimental animals, and since our previous studies had shown ICAM-1 activation at 2 weeks after AAV2-SYN administration [13], we used this earlier time point for these mRNA analysis. Groups of 6 of WT or FcγR-/- mice were injected stereotaxically with AAV2-SYN or AAV2-GFP into the right SN. The brain tissue was processed for quantitative PCR (QPCR). All values were normalized against glyceraldehyde-3-phosphate dehydrogenase (GAPDH) mRNA measured in the same samples.

**Figure 3:**
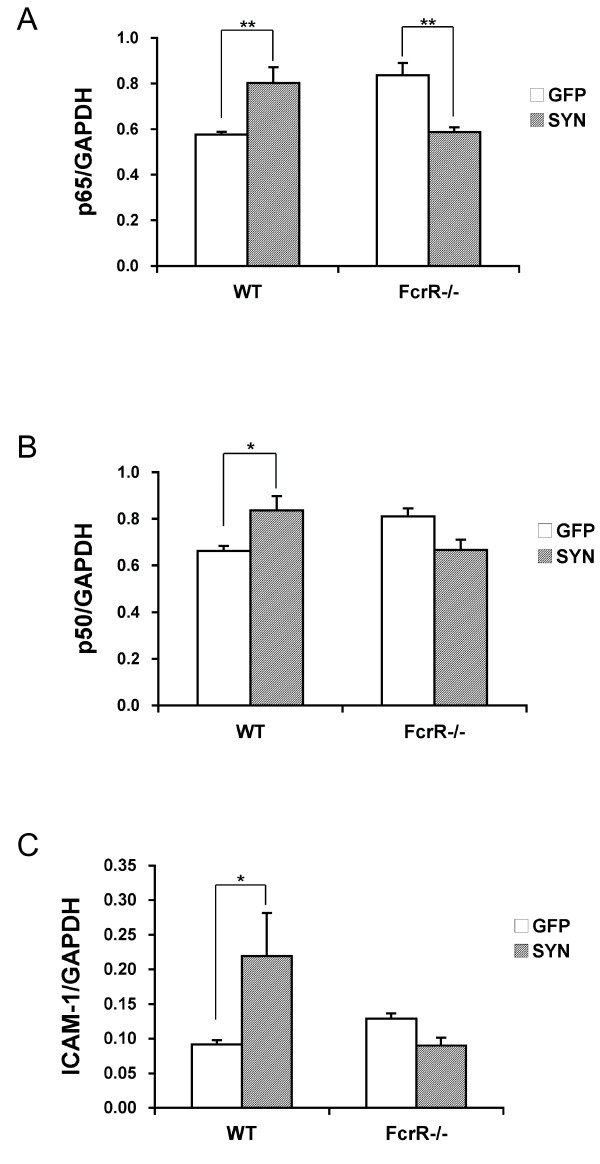
**Effect of α-SYN over-expression on the transcription of NF-κB components and the pro-inflammatory mediator in wild-type and FcγR-/- mice at two weeks**. There was significant increase in the mRNA level of A) NF-κB p65, B) NF-κB p50, C) intercellular adhesion molecule 1(ICAM-1) in the WT SYN group compared to the WT GFP group. **p < 0.01, *p < 0.05 WT GFP vs WT SYN; and the mRNA level of NFκB p65 was decreased in FcγR-/- SYN mice compared with FcγR-/- GFP mice. **p < 0.01 FcγR-/- SYN vs FcγR-/- GFP. One-way ANOVA with Fisher PLSD post hoc test, GAPDH, glyceraldehyde-3-phosphate dehydrogenase.

We first examined the levels of the NF-κB p65 and p50 mRNAs. In the wild-type mice, we observed that overexpression of human α-SYN resulted in elevated abundance of both NFκB p65 and p50 mRNA in the SN compared with wild-type AAV2-GFP treated mice. In contrast, the mRNA level of NFκB p65 was decreased, rather than increased, in the FcγR-/- SYN mice compared with FcγR-/- GFP mice. In addition, there was trend towards reduction of NFκB p50 mRNA in FcγR-/- SYN mice compared to FcγR-/- GFP mice, which did not reach statistical significance (Figure 3A and 3B).

As a measure of the effect of NF-κB on downstream inflammatory mediators, we studied the abundance of the mRNA for intercellular adhesion molecule 1 (ICAM-1), which contains a consensus sequence for binding of NF-κB p65 [26]. We had previously observed that ICAM-1 was increased by AAV2-SYN at both 2 weeks and 4 weeks post-injection in wild-type mice [13]. In these experiments we confirmed the effect of AAV2-SYN on ICAM-1 mRNA levels in wild-type mice, which led to a more than two fold induction. In contrast, we observed no evidence for the activation of ICAM-1 transcription in the FcγR-/- mice, with similar levels of ICAM-1 mRNA in both FcγR-/- GFP and FcγR-/- SYN groups (Figure 3C).

### Microglial activation is attenuated in FcγR-/- mice

**Figure 4:**
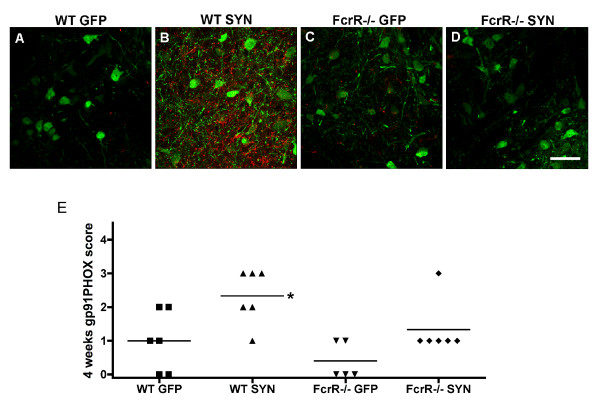
**Effect of α-SYN over-expression on microglial activation in wild-type and FcγR-/- mice**. A-D) At four weeks after AAV injection, SN sections of wild-type and FcγR-/- mice over-expressing α-SYN or GFP were double-stained for gp91PHOX (red) and SYN/GFP (green). Wild-type mice expressing α-SYN revealed significantly increased immunoreactivity for gp91PHOX compared to the other three groups. gp91PHOX immunoreactivity was localized to microglia, which appear to be close to α-SYN-expressing neurons (scale bar = 50 μm). E) Scoring of microglial activation using a rating scale revealed significant microglial activation in wild-type mice expressing α-SYN compared to those expressing GFP. This difference was not observed in FcγR-/- mice expressing SYN or GFP. *p < 0.05, WT-SYN vs WT-GFP, Mann-Whitney U test.

Microglial activation was examined at four and twenty-four weeks following AAV2-SYN or AAV2-GFP administration using gp91PHOX as a marker [27]. At four weeks post-injection, we noticed a significant increase in gp91PHOX immunoreactivity in the wild-type animals treated with AAV2-SYN compared to those treated with AAV2-GFP (Figure 4). In α-SYN-expressing wild-type mice, activated microglia were noticed predominantly on the injected side. These cells were distributed rostro-caudally from the injection site close to the SN and also seen in the surrounding areas. In the FcγR-/- mice, neither the AAV2-SYN nor the AAV2-GFP induced any appreciable increase in gp91PHOX immunoreactivity except for some staining of gp91PHOX along the needle tract present in all the groups; this was presumed to be related to local trauma and was not considered for quantification.

In the 24-week groups, we use a different staining method because of the intrinsic autofluoresence present in older animals. This method is less sensitive than the fluorescent method used at the earlier time point, and thus the data from the 24 week observations are not directly comparable to those obtained at the earlier time points. We found that after 24 weeks, the intensity of gp91PHOX staining microglia was modest and similar in AAV2-SYN injected wild-type mice as well as FcγR-/- mice. There was a trend towards increased gp91PHOX positive microglia in both wild-type mice and FcγR-/- mice treated with AAV2-SYN compared to their AAV2-GFP treated counterparts, but the difference in gp91PHOX between SYN and GFP groups was not statistically significant.

### α-SYN-induced dopaminergic neurodegeneration is attenuated in FcγR-/- mice

To examine the effect of FcγR deficiency on α-SYN-induced neurodegeneration, we examined animals 24 weeks following AAV2-SYN or AAV2-GFP administration. Sequential sections through the midbrain were stained for tyrosine hydroxylase (TH) and the TH-positive dopamine neurons were quantified using unbiased stereology.

**Figure 5:**
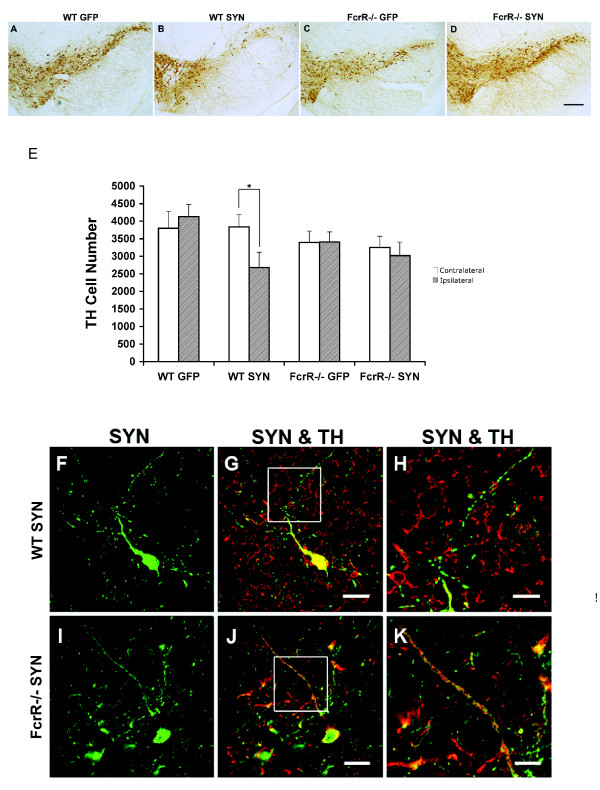
**Effect of α-SYN over-expression on dopaminergic neurodegeneration in wild-type and FcγR-/- mice**. A-D) The SN of wild-type and FcγR-/- mice expressing α-SYN or GFP was stained for tyrosine hydroxylase 24 weeks after treatment. The images depict a representative sample of the injected side of the SN of the four treatment groups. E) Counts of TH positive neurons using unbiased stereology. The wild-type mice treated with AAV2-SYN revealed a significant reduction in dopamine neuron count in the injected side compared to the un-injected side. No significant loss of dopamine neurons was observed in wild-type mice treated with AAV2-GFP and FcγR-/- mice expressing GFP/SYN. *p < 0.05, one-tailed t test, ipsilateral vs contralateral in WT SYN mice. F-K) Representative neurite samples of WT AAV2-SYN and FcγR-/- AAV2-SYN mice. The α-SYN(green)-expressing neuron in WT mice has little TH (red) staining in the beaded degenerating neurite, while FcγR-/- mice are protected from neuritic change. Panel H and K are high-magnification images of the neurites in the square of panel G and J respectively. Scale bars: panels A, B, C, D bar = 200 μm; panels F, G, I, J bar = 25 μm; panels H, K bar = 6 um.

Visual examination revealed a consistent pattern of loss of TH immunoreactive neurons in the injected side compared to the non-injected side in wild-type mice treated with AAV2-SYN compared to the other three groups (Figure 5A-D). In the wild-type mice treated with AAV2-SYN, many neurons presented with very weak immunostaining for TH, and degenerating neurites were apparent, characterized by beading and loss of TH staining even when strong TH staining was still evident in the cell body (Figure 5F-H). Stereological analysis revealed a 27% reduction in the number of TH-positive neurons on the injected side compared to the contralateral side in the wild-type AAV2-SYN treated mice (P < .03).

In contrast, the administration of AAV2-SYN to FcγR-/- mice resulted in no apparent loss of TH staining, and no degenerating neurites were appreciated (Figure 5I-K). Quantitative analysis with stereology revealed no significant reduction in TH-positive neuronal count in FcγR-/- mice expressing α-SYN compared to those expressing GFP (Figure 5E).

## Discussion

In these studies, we have found that AAV2-mediated over-expression of human α-SYN in mouse SN triggers NF-κB activation, with transcriptional induction of the p65 and p50 components, as well as induction of the pro-inflammatory mediator ICAM-1. There is microglial activation and marked accumulation of p65 protein in microglia. Deficiency of FcγR blocks the transcriptional induction of p65, p50, and ICAM-1, prevents microglial p65 accumulation. Moreover, α-SYN-induced microglial activation and dopaminergic neurodegeneration are attenuated in FcγR-/- mice.

We employed an AAV2 mouse model of Parkinson's disease in our study, which we have characterized previously [13]. Vector strategies provide both targeted delivery and high levels of transgene expression. The AAV vector encodes no viral proteins, and the vector itself has little or no immunogenicity or toxicity; indeed, we observed no significant immunological activation in our control animals, despite strong expression of the encoded GFP protein. In the mouse AAV model, microglial activation and neuroinflammation are early events, while loss of dopaminergic neurons occurs later, after more than 3 months [28]. This profile differs from similar AAV models in rats, where the degenerative process appears to be more rapid [29]. Both of these AAV-mediated approaches replicate the dopaminergic neurodegeneration which is the signature characteristic of human PD. Transgenic animals expressing α-SYN, in contrast, rarely show dopaminergic cell loss, or only at very advanced ages [30]. At the other extreme, neurotoxin-induced PD models, such as the mouse MPTP model, lead to rapid necrosis of dopamine neurons with oxidative damage to DNA, lipids, and proteins [31,32]. There is prominent neuroinflammation after MPTP treatment, but the relevance of this to the gradual process of cell injury in human PD is certainly open to question.

Our data point to a central role for FcγR proteins in mediating α-SYN-induced neuroinflammation. In the mouse, the classic FcγRs are well characterized and include FcγRI, FcγRIIB and FcγRIII. Both FcγRI and FcγRIII are multi-chain complexes composed of a single ligand-binding α-chain and a homodimer of common γ-chains that mediates intracellular signaling through an immuno-receptor tyrosine-based activation motif (ITAM) in the cytoplasmic domain [33]. The FcγR-/- mice that we used in our studies are deficient in the γ-chain subunit of the FcγRI and FcγRIII, leading to approximately 20% of normal levels of FcγRI expression, and total lack of FcγRIII expression. Thus, our data do not allow us to distinguish between effects mediated by FcγRI or FcγRIII, and it is possible that either or both of them are involved in the α-SYN-induced neuroinflammation. It is also important to note that although the FcγR-/- mice have been backcrossed to the genetic background of C57BL/6 mice, they do exhibit immune system defects. This likely accounts for the baseline differences we observed in the expression of NF-κB components and ICAM-1 mRNA in the FcγR-/- mice compared with wild-type mice (Figure 2 and 3). We also observed that the FcγR-/- mice had greater baseline abundance of p65 in the nuclear fraction (Figure 2J), likely also a consequence of altered immunity in these animals. Interestingly, they do not appear to have an increase in cytoplasmic p65 at baseline, as evidenced by the low level of p65 staining observed using immunohistochemistry (Figure 2C). The significance of this disparity is uncertain; nuclear p65 is clearly linked to transcriptional effects, but the potential activities of cytoplasmic p65 are much less clear. Because of these baseline disparities between control and FcγR-/-animals, we have based our conclusions on comparisons of the results of AAV2-GFP and AAV2-SYN treatment within the WT or FcγR-/- groups.

In this study, we observed a significant role of NF-κB in α-SYN-induced neuroinflammation and neurodegeneration. In unstimulated cells, NF-κB is bound by the inhibitor IκB which sequesters NF-κB in the cytoplasm. Activation of NF-κB is initiated by the signal-induced degradation of IκB proteins. This occurs primarily via the activation of IκB kinase (IKK), which can phosphorylate IκB at two conserved N-terminal Ser residues. Subsequent ubiquitination and degradation of the inhibitor results in liberation of the heterodimeric NF-κB complexes, which are able to migrate into the nucleus and to regulate gene expression [20]. In mammals, the most abundant NF-κB complex is p65/p50 [34]. In our model, we observed increased mRNA for both p65 and p50, but only the p65 protein exhibited prominent accumulation in microglia. The reason for the relative lack of accumulation of p50 is uncertain, but it might be related to differences either in mRNA translation or protein degradation. It is also possible that in this model, p65 has partners other than p50, such as p52 [35]. The data on ICAM-1 provide evidence that NF-κB signaling was indeed activated, as this gene is strongly regulated by the binding of NF-κB to an intronic site [26]. The transcriptional activation of p65 and p50, the accumulation of p65 protein, and the induction of ICAM-1 were all attenuated in the FcγR-/- animals.

Our observations also suggest that the lack of FcγR protein attenuates neurodegeneration and loss of TH-positive neurons, but it is important to consider this finding in the context of the limitations of the model system. In wild-type animals, AAV2-SYN led to the appearance of degenerating neurites, and these were greatly reduced in the FcγR-/- animals. To assess the differences quantitatively, we used unbiased stereology to count surviving TH-positive neurons in the SN. We observed loss of TH-positive cells in the wild-type animals after AAV2-SYN but, as in most rodent α-SYN models of PD, the degree of loss induced by α-SYN overexpression was modest (27%) and there was considerable variability (SD = 22). We did not observe loss of TH cells in the FcγR-/- animals, but the statistical power of our experiment to detect a reduction in the size of this already modest effect is limited; a power calculation suggests that the group sizes employed here provide less than 80% power to detect this degree of cell loss. Achieving 95% power would require group sizes of at least 24, a total of nearly 100 animals, which is impractical. Although the data on TH neuron number we obtained are consistent with the qualitative assessment of neuritic change and our other observations, we cannot exclude the possibility of a Type II error and this particular finding requires further evaluation in models with more robust baseline neurodegeneration.

The AAV2 model is very different from MPTP neurotoxin models of PD, but role of NF-κB signaling in the two types of models is remarkably similar. In MPTP treated mice, there is also marked accumulation of p65 in microglia, and activation of NF-κB was detected within the SN by electrophoretic mobility shift assay (EMSA) [22]. Cytokine expression was also increased in MPTP-intoxicated mice [22]. Injection of NEMO-binding domain (NBD) peptide, which inhibits NF-κB activation, suppressed microglial activation and cytokine production, protected both the nigrostriatal axis and neurotransmitters, and improved motor functions in MPTP-intoxicated mice [22]. In the AAV model, the evidence linking NF-κB to neurodegeneration is at present more circumstantial; we have observed that deletion of FcγR reduces both NF-κB activation and neurodegeneration, but it does not establish that NF-κB is solely responsible for these effects, and it is possible that FcγR deletion also alters other signaling pathways. In this context, studies using pharmacological inhibitors of NF-κB in the AAV model would be very valuable.

## Conclusion

**Figure 6:**
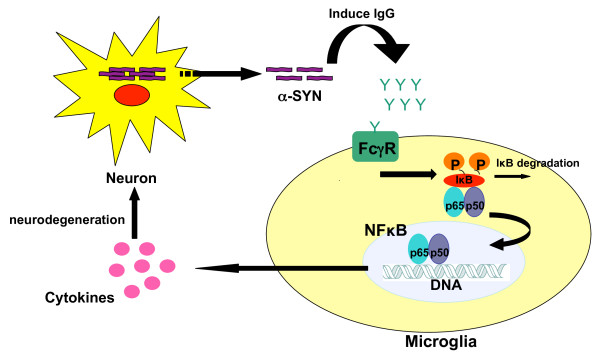
**α-SYN induced neuroinflammation model**. Over-abundance of α-SYN leads to the expression of a specific antigen, which induces IgG generation. The binding of IgG and FcγR on the surface of microglia results in the downstream signaling pathways, in which NF-κB is the key transcription factor, and causes microglial activation. Microglial activation results in a flooding of surrounding tissue with a variety of neurotoxic substances such as cytokines that ultimately injure neurons in the SN.

In sum, our data provide evidence that FcγR proteins provide an important interface between the adaptive immune response, specifically the humoral response, and the neurodegenerative processes triggered by α-SYN over-expression. The humoral response is generated by excess α-SYN, although the nature of the specific antigen is still uncertain. In MPTP neurotoxin models, there is evidence, which suggests that nitrated forms of α-SYN may be responsible, but whether this is also the case in the AAV model (and the human disease) is unclear. We propose that the binding of this induced IgG to FcγR on the surface of microglia results in the downstream signaling pathways, in which NF-κB is the key transcription factor, and causes microglial activation and cytokine production that ultimately injures neurons in the SN (Figure 6). This hypothesis suggests that inhibition of either FcγR signaling or specific intracellular signal transduction events downstream of FcγR-IgG interaction, such as NF-κB activation, may be viable therapeutic strategies to slow or prevent the progression of human PD.

## Abbreviations

α-SYN: alpha-synuclein; AAV2: adeno-associated virus serotype 2; ANOVA: analysis of variance; DA: dopamine; FcγR: Fc gamma receptor; GFP: green fluorescent protein; IgG: immunoglobulin G; MPTP: 1-methyl 4-phenyl 1,2,3,6-tetrahydropyridine; NADPH: nicotinamide adenine dinucleotide phosphate; NF-κB: Nuclear factor kappa-light-chain-enhancer of activated B cells; PD: Parkinson's Disease; SN: substantia nigra pars compacta; 6-OHDA: 6-hydroxydopamine; TH: tyrosine hydroxylase.

## Competing interests

The authors declare that they have no competing interests.

## Authors' contributions

SC carried out the studies for NFκB p65 and p50, quantitative PCR, and data analysis, participated in the design of the study and drafted the manuscript. ST participated in the design of the study, carried out the studies for gp91PHOX and stereology for dopamine neurons. DS participated in the design of the study, carried out the immunostaining scoring and helped to draft the manuscript. All authors read and approved the final manuscript.
